# Supplementary figures and images for: Networks of VTA Neurons Encode Real-Time Information about Uncertain Numbers of Actions Executed to Earn a Reward
Source: Front Behav Neurosci. 2017 Aug 8;11:140. doi: 10.3389/fnbeh.2017.00140 (PMC5550723; doi:10.3389/fnbeh.2017.00140)

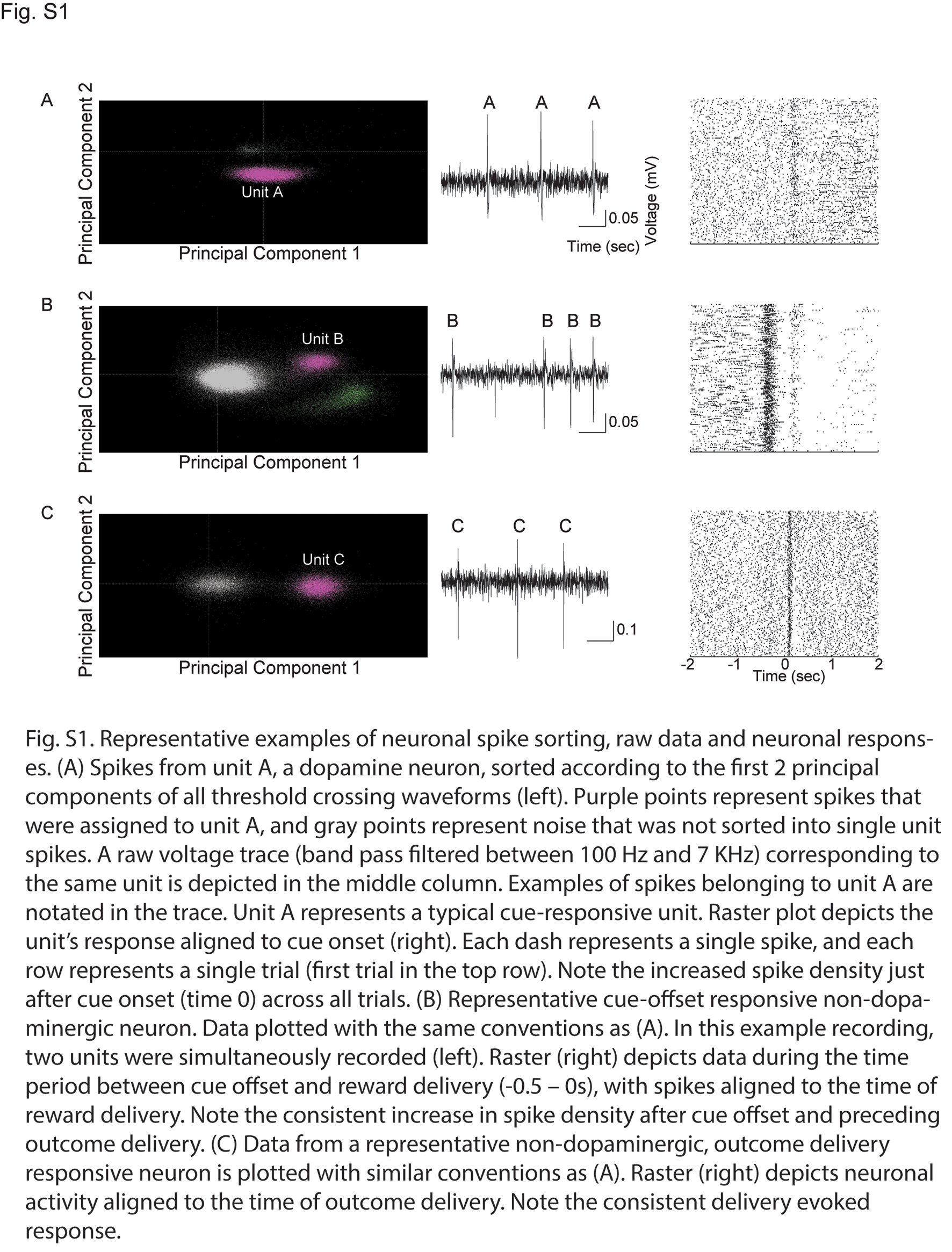

Supplement: Figure S1 — Representative examples of neuronal spike sorting, raw data and neuronal responses. (A) Spikes from unit A, a dopamine neuron, sorted according to the first 2 principal components of all threshold crossing waveforms (left). Purple points represent spikes that were assigned to unit A, and gray points represent noise that was not sorted into single unit spikes. A raw voltage trace (band pass filtered between 100 Hz and 7 KHz) corresponding to the same unit is depicted in the middle column. Examples of spikes belonging to unit A are notated in the trace. Unit A represents a typical cue-responsive unit. Raster plot depicts the unit's response aligned to cue onset (right). Each dash represents a single spike, and each row represents a single trial (first trial in the top row). Note the increased spike density just after cue onset (time 0) across all trials. (B) Representative cue-offset responsive non-dopaminergic neuron. Data plotted with the same conventions as (A). In this example recording, two units were simultaneously recorded (left). Raster (right) depicts data during the time period between cue offset and reward delivery (−0.5 to 0 s), with spikes aligned to the time of reward delivery. Note the consistent increase in spike density after cue offset and preceding outcome delivery. (C) Data from a representative non-dopaminergic, outcome delivery responsive neuron is plotted with similar conventions as (A). Raster (right) depicts neuronal activity aligned to the time of outcome delivery. Note the consistent delivery evoked response. [file Image1.TIF]
